# Report of a Case of Opisthotonos Cured by Ice

**Published:** 1864-12

**Authors:** Edward Howard

**Affiliations:** Red Hill, Surrey


REPORT OF

### A CASE OF OPISTHOTONOS CURED BY ICE.

By EDWARD HOWARD, M. R. C. P, L.

This case seems worthy of record in a medico-legal point of view, as, under many circumstances, poisoning by strychnia would have been strongly suspected.

On the 13th of August, in the afternoon, I was summoned to attend Mr. C——, aged twenty-five, of bilious aspect and nervous temperament. He is a foreman in a draper's shop, but much out of doors as an agent collecting orders. About two years ago he was under my care with marked hysteria, which diagnosis was subsequently confirmed by other physicians who saw him. From that he recovered by a liberal diet and a general mineral-tonic treatment. He has enjoyed good health during the last eighteen months, although I do not doubt he has had much anxiety from failure in trade.

On arriving, I found my patient lying over the back of a chair, his shoulders and legs being supported by four attendants. His body was drawn backward and arched; attempts to put him straight caused a fearful increase of pain, which he described as like having a testicle pressed strongly. There was also a sense of suffocation from contraction of the abdominal and intercostal muscles; heart's sounds and action normal; pulse feeble and quick (72.) I had him placed on a couch, with a sofa pillow under his back as a support. He was perfectly sensible, and had had no fit or loss of consciousness. A few hours before, he had a dull pain in the spine between the shoulders; this increased, and he found himself unable to speak to a customer he was serving. He went to the back of the house to get fresh air, and then was suddenly drawn back by the tonic spasm in which I found him. Tongue moist and clean, and the bowels regular. He had received no wound or injury of any kind, nor taken anything that could have disagreed with him. There was no sickness or nausea. Ordered a light nutritious diet, and five grains of sesquicarbonate of ammonia in infusion of gentian every fourth hour.

Aug. 14th.—Morning: Much the same; not worse, but no definite amelioration. Ordered ice to be constantly applied along the spine, and to continue medicine. Evening: Better; the spasm less violent, and the breathing unimpeded. To continue the ice, and take five grains of citrate of iron and quinine every sixth hour.

15th.—Still improving. The opisthotonos has vanished; only tenderness along the dorsal spine remains, and slight spasmodic pains on sudden movements of the body.

I need only add that the progress to entire recovery was rapid, and hitherto there has been no sign of a relapse.

Red Hill, Surrey, Sept. 10th, 1864.

---

Effects of Ergot of Wheat.—The following fact deserves to be noticed since it may become the starting point of researches and may be of useful therapeutical application. No one has, so far as I know, mentioned the fact observed by Dr. Poyet, suppression of milk under the influence of the habitual use of bread containing a notable proportion of ergot of wheat. This accident has taken place in six nurses who had eaten a considerable quantity of such bread. It should all the more fix the attention of practitioners that it has been also observed, at the same time, by Dr. Commarmond, and that it seemed destined, in consequence, to find its proper place in the symptomatology of ergotism. I may add that in the above circumstances the substitution of good wheat bread to the diseased one sufficient to avert this accident and restore the milk secretion.—*Med. and Surgical Reporter.*

